# Public awareness and perception of robotic-assisted surgery: a cross-sectional analysis of sociodemographic influences

**DOI:** 10.3389/fpubh.2025.1662689

**Published:** 2025-10-07

**Authors:** Husna Irfan Thalib, Khadijah Tahir, Ayesha Shrouq Amin, Zahra Hussein Alabdrabalrasol, Sarah Kaleem Ather, Bader Mahmoud Almurad, Sara Ahmed Al Nawajha, Ahmed A. Elshora, Mohammed Ridha Algethami, Noor Ahmad Shaik, Salem Sroor Al Bagmi

**Affiliations:** 1 College of Medicine and Surgery, Batterjee Medical College, Jeddah, Saudi Arabia; 2 Department of Surgery, General Medicine Program, Batterjee Medical College, Jeddah, Saudi Arabia; 3 Department of Family and Community Medicine, College of Medicine, University of Jeddah, Jeddah, Saudi Arabia; 4 Department of Genetic Medicine, Faculty of Medicine, King Abdulaziz University, Jeddah, Saudi Arabia; 5 Department of Health Information Management, Prince Sultan Military College of Health Sciences, Dammam, Saudi Arabia

**Keywords:** robotic-assisted surgery, public awareness, Saudi Arabia, healthcare adoption, perception

## Abstract

**Background:**

Robotic-assisted surgery (RAS) is increasingly prevalent in Saudi Arabia, yet public awareness and acceptance remain inconsistent. This study aimed to evaluate sociodemographic factors influencing familiarity and perception of RAS among the Saudi population.

**Methods:**

An analytical cross-sectional survey was conducted between November 2024 and April 2025 among 681 adults across all major regions of Saudi Arabia using a convenience sampling strategy. A validated, bilingual (Arabic/English) questionnaire assessed RAS awareness, safety perceptions, and concerns. Descriptive statistics summarized responses, chi-square tests explored associations, and binary logistic regression identified predictors of awareness and perception. Odds ratios (ORs) and 95% confidence intervals (CIs) were reported, with significance set at *p* < 0.05.

**Results:**

Overall, 27.2% of participants reported familiarity with RAS, and 59.5% expressed safety concerns. Females demonstrated higher familiarity than males (OR = 1.45, 95% CI = 1.05–2.01, *p* = 0.02), while males were more likely to perceive RAS as unsafe (OR = 0.50, 95% CI = 0.34–0.74, *p* = 0.001). Saudis were significantly more familiar than non-Saudis (OR = 1.75, 95% CI = 1.16–2.64, *p* = 0.008). Participants from the Southern region were more likely to perceive RAS as safe (OR = 2.00, 95% CI = 1.22–3.27, *p* = 0.006).

**Conclusion:**

This study identifies demographic predictors of awareness and perception of RAS, underscoring the need for targeted educational campaigns, public health messaging, and integration of RAS into medical curricula. Such strategies can improve trust, reduce misconceptions, and facilitate equitable adoption of advanced surgical technologies in Saudi Arabia.

## Introduction

Robots have been used in different fields since the 1960’s for various tasks allowing faster, more precise, and consistent execution. Recently, their use has expanded into the surgical field assisting surgeons in conducting minimally invasive surgeries (1, 2). Robotic assisted minimally invasive surgery allows less experienced laparoscopic surgeons to perform accurate and higher quality surgeries (3, 4). Robotic surgery systems offer advantages over traditional laparoscopic techniques including enhanced three-dimensional visualization, depth perception, a broader range of motion, and the elimination of hand tremor issues (5, 6). Previous studies have shown that robotic-assisted surgery aids in performing precise surgical procedures, resulting in reduced lengths of hospital stays after surgery and improved patient outcomes with fewer complications in comparison to open surgery (3, 4). Despite these advancements, the use of robotic-assisted surgery (RAS) remains low in Saudi Arabia compared to Western countries. Understanding how the public views robotic-assisted surgery and developing strategies to promote its benefits is crucial to address this issue. Most existing research has focused on the viewpoints of either patients’ or medical staff’s regarding RAS leaving a gap in knowledge about the general public’s understanding, attitude, and awareness of RAS which caused misconceptions. This study aims to fill that gap by assessing the public’s perception of RAS in Saudi Arabia. While awareness of robotic surgery is gradually increasing in Saudi Arabia, concerns about robot malfunction, surgical errors, and surgeon competency persist (7). Factors influencing awareness include gender, education, income, occupation, computer literacy, and technology comfort (7). To facilitate successful integration of RAS in surgical practices, it is crucial to address these concerns, enhance public comprehension, and awareness to promote informed decision-making (7, 8).

In Saudi Arabia, robotic-assisted surgery is still at an early stage and remains largely confined to tertiary care hospitals. Its gradual introduction aligns with the broader healthcare modernization agenda outlined in Vision 2030, which emphasizes digital transformation and the integration of advanced medical technologies. Despite these initiatives, public familiarity with and acceptance of RAS have not kept pace. Misconceptions and limited understanding may undermine trust and slow the equitable adoption of such innovations, even in the face of significant national investment. Most previous research in the Kingdom has examined the perspectives of patients attending surgical clinics or medical students, leaving the general public’s views largely unexplored. This gap is important because public perceptions directly affect informed consent, confidence in healthcare providers, and willingness to undergo new surgical approaches. Addressing it is therefore critical. The present study aims to assess public awareness of robotic-assisted surgery, explore attitudes toward its safety and effectiveness, and identify the sociodemographic factors that shape these perceptions.

## Methods

### Study design

This quantitative analytical cross-sectional study was conducted through a survey- based questionnaire over a period of 6 months, from November 2024 to April 2025. The target population for this study consisted of adults residing in the major cities from different regions of Saudi Arabia.

### Sampling method

A non-probability convenience sampling method was employed. Dedicated data collectors distributed the survey link primarily through WhatsApp group chats, with the aim of reaching participants from diverse regions and backgrounds across Saudi Arabia. Adults aged 18 years or older, residing in Saudi Arabia, and able to complete the questionnaire in Arabic or English were eligible to participate. Exclusion criteria included individuals younger than 18 years, non-residents, and healthcare professionals specializing in robotic surgery. This approach was considered the most practical within the study’s time and resource constraints. While convenience sampling carries inherent limitations in terms of representativeness, the use of multiple group networks and regional coverage helped ensure participation from a wide range of demographic groups.

### Study subjects

This study includes all adults who are aged 18 and above, living in different regions of Saudi Arabia, regardless of their nationality. Only those who could understand and complete the questionnaire in either Arabic or English were allowed to participate. An informed consent was taken from the participants. Those with a general interest or involvement in healthcare were included too. However, non-residents living elsewhere (other than Saudi Arabia), individuals under 18, people with cognitive impairments or language barriers, and healthcare professionals specializing in robotic surgery were excluded from this study. Additionally, participants who did not consent or chose to withdraw from the study were excluded to maintain ethical standards. Socioeconomic status (SES) was assessed using monthly household income, categorized into three brackets (<6,000 SAR, 6,000–15,000 SAR, and >15,000 SAR) based on local labor market standards, together with highest educational attainment. Regional residence was classified into Central, Eastern, Western, Northern, and Southern regions; within this framework, the Central and Western regions are predominantly urban, while the Northern and Southern regions include largely rural populations. Health literacy was not measured directly but was approximated through proxies including education level and self-reported technological literacy, which have been shown to correlate with health information-seeking behavior in the Saudi context. These categorizations were chosen to reflect national demographic groupings and align with health policy priorities.

### Sample size

The sample size was calculated using Cochran’s formula for cross-sectional studies (9):


$$n=(Z^{2}\times p\times (1-1p))/E^{2}$$

where *Z* = 1.96 for a 95% confidence level, *p* = 0.50 to maximize variability, and *E* = 0.05 margin of error. Based on the adult population of Saudi Arabia (34.6 million) (10), the minimum required sample size was 385 participants. To account for potential non-responses/incomplete responses, we adjusted the sample size by 20%, increasing it to 462 participants. This sample size ensures sufficient responses to detect significant differences in awareness and attitudes, aligning with standard practices in population-based health research. This approach also ensured that the study results will be both statistically reliable and generalized to the adult population of Saudi Arabia.

Study tool: The questionnaire used in this study was developed based on previously published instruments and consisted of 29 items organized into four sections: consent, socio-demographic data, awareness of robotic-assisted surgery (RAS), and attitudes/perceptions toward RAS. The items were initially formulated in English and then translated into Arabic to ensure clarity and cultural relevance. The translation process involved two bilingual experts, followed by review and harmonization by a panel of specialists to resolve discrepancies. A backward translation into English was subsequently performed to verify accuracy. To further refine the tool, a pilot test was conducted with 30 participants to assess readability and comprehension, after which minor adjustments were made based on participant feedback. For the purposes of this study, “awareness” was defined as whether a participant had heard of RAS and could identify at least one of its applications, while “perception” referred to views regarding safety, cost, training requirements, and overall acceptance of RAS. Reliability testing of the final Arabic version demonstrated good internal consistency, with a Cronbach’s alpha of 0.81.

### Data collection

The data were collected digitally using a structured validated questionnaire through Microsoft forms, which was randomly shared online to participants. The questionnaire was designed to capture both the level of awareness and attitude of participants towards robot- assisted surgery. The questionnaire included sections on demographic information, knowledge of robot-assisted surgery, perceived benefits and risks, and overall attitudes towards the adoption of this technology in healthcare.

### Ethical considerations

To ensure that the study fulfills the criteria relevant for ethical standards, the study has been approved by the institutional review board (IRB) of Batterjee Medical College (IRB approval code no. RES-2024-0079). Ethical guidelines were strictly followed by informing the participants that their participation is completely voluntary. A consent checkbox was included in the beginning of the survey to ensure that they have willingly agreed to participate. All responses were collected anonymously, in order to ensure confidentiality. The data collected was used for research purposes entirely, and no personal information or identity of the participants has been reported in this research.

### Data analysis

The IBM Statistical Package for Social Studies (SPSS) Version 26 was used for the statistical analysis. Descriptive statistics were used to summarize demographic information and the overall awareness and attitude towards robot-assisted surgery. Chi-square tests were also used to assess associations between demographic variables and the level of awareness. The significance level was at a *p*-value less than 0.05 for all statistical tests. Binary logistic regression was used to identify predictors of high levels of awareness and familiarity. Odds ratios (ORs) and 95% confidence intervals (CIs) were reported. Statistical significance was set at *p* < 0.05.

**Figure 1:**
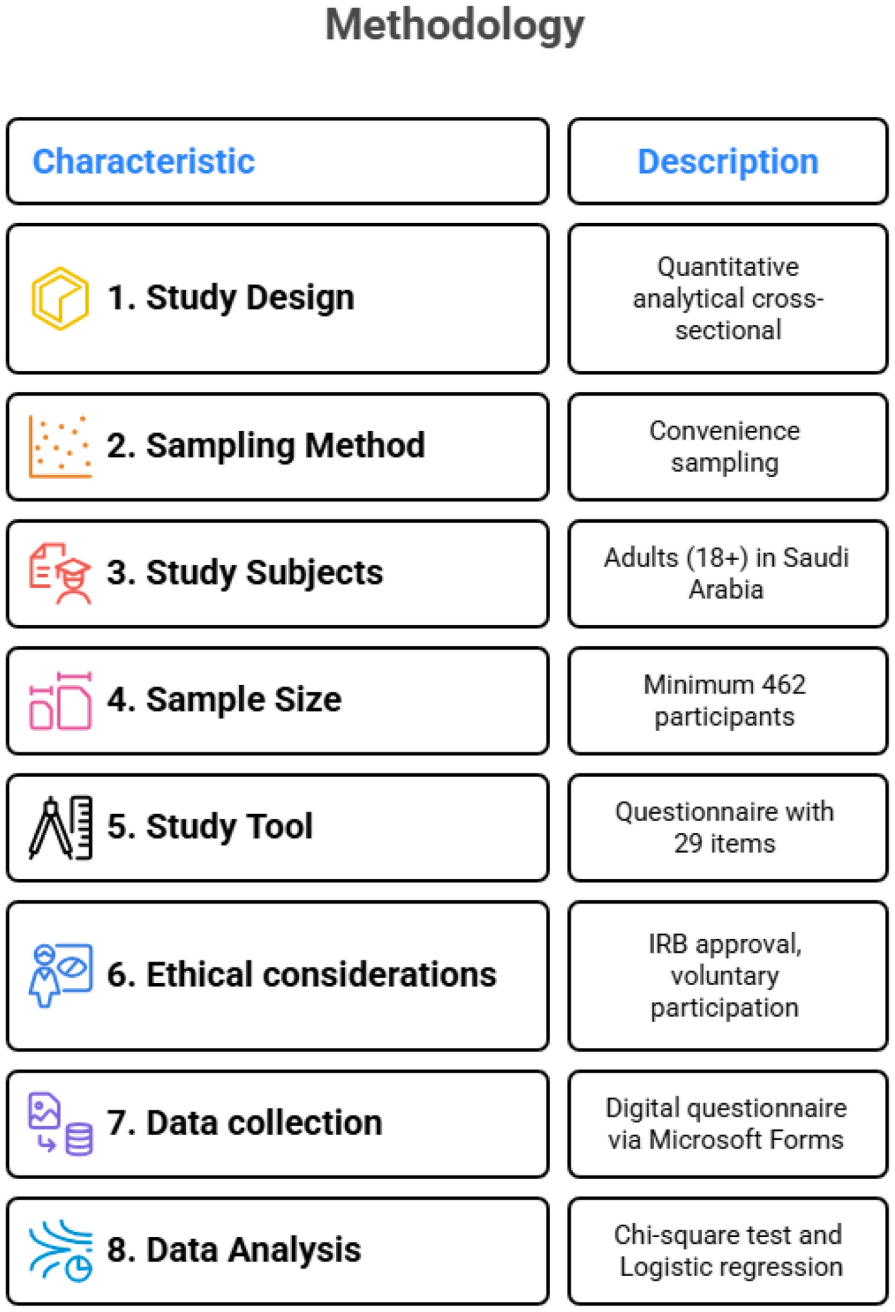
Flowchart demonstrating the methodology.

The methodological framework adopted in this cross-sectional study is illustrated in Figure 1.

## Results

A total of 681 participants were included in this study. The largest age group was 35–44 years (28.9%), while only a small percentage (0.7%) were 65 years or older as shown in Table 1. More females (59.5%) participated than males (40.5%). A majority of the participants were Saudi nationals (70.6%), with most living in the Western (32.5%) and Southern (29.7%) regions of Saudi Arabia. More than half of the participants (52.1%) were married, and nearly half (49.5%) held a bachelor’s degree. In terms of employment, 36.0% worked outside the healthcare field, while 26.1% were students. Most participants (66.5%) were considered technologically literate, and the majority (54.0%) spent 5–9 h per day using electronic devices.

**Table 1:** Socio-demographic characteristics of study participants.

Variable	Frequency (N)	Percentage (%)
Age		
18–24	185	27.2
25–34	133	19.5
35–44	197	28.9
45–54	129	18.9
54–64	32	4.7
65 and above	5	0.7
Gender		
Males	276	40.5
Females	405	59.5
Nationality		
Saudi	481	70.6
Non-Saudi	200	29.4
Region in Saudi Arabia		
Central	89	13.1
Eastern	132	19.4
Western	221	32.5
Northern	37	5.4
Southern	202	29.7
Marital status		
Divorced	23	3.4
Married	355	52.1
Single	295	43.3
Widowed	8	1.2
Education level		
Bachelor’s degree	337	49.5
Diploma	115	16.9
High school or lower	149	21.9
Postgraduate degree	80	11.7
Work field		
Health care field	103	15.1
Not healthcare field	245	36
Not working	124	18.2
Retired	31	4.6
Student	178	26.1
Monthly Income		
Below 6,000	318	46.7
6,000–15,000	231	33.9
Above 15,000	132	19.4
Technological literacy		
Competent	168	24.7
Literate	453	66.5
Illiterate	60	8.8
Hours spent on an electronic device		
Less than 4 h	161	23.6
5–9 h	368	54
10–15 h	124	18.2
More than 15 h	28	4.1
TOTAL	681	100

**Figure 2:**
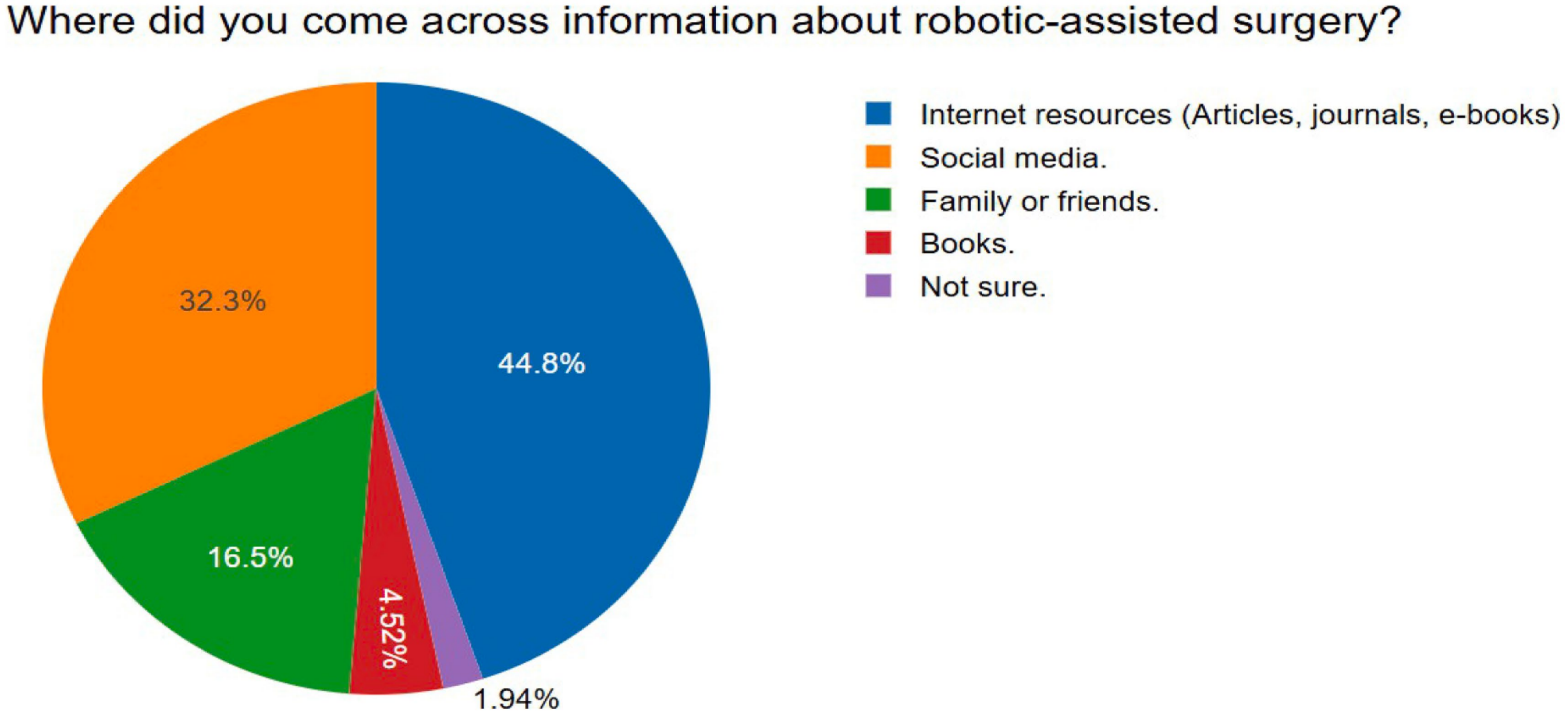
Sources of public awareness on robotic-assisted surgery.

The pie chart illustrates the distribution of sources through which respondents reported acquiring information about robotic-assisted surgery (Figure 2). The majority of participants (44.8%) indicated that they obtained information from internet-based resources, including articles, journals, and e-books. Social media was identified as the second most prevalent source, accounting for 32.3% of responses. Information from family or friends constituted 16.5% of the total, while a smaller proportion of respondents cited books (4.52%) as their source. A minority (1.94%) reported being unsure of where they had encountered information on the topic.

Table 2 demonstrates the results of a chi-square analysis that examined which socio- demographic factors were linked to familiarity with robotic-assisted surgery. The results show that age, nationality, region, marital status, and work field were significantly related to familiarity (*p* < 0.05). Younger participants (18–24 years) and those working in the healthcare field were more likely to be familiar with robotic-assisted surgery. Saudi participants reported greater familiarity compared to non-Saudis. There were also differences based on region, with participants from the Central and Western regions showing higher familiarity. Marital status played a role, as single participants were more familiar with robotic-assisted surgery than married or widowed individuals.

**Table 2:** Chi-square analysis of socio-demographic factors influencing familiarity with robotic assisted surgery among study participants.

Familiarity with robotic assisted surgery among study participants					
Variable		Total (%)	Yes	No	*p*-value
Age	18–24	185 (27.2)	109	76	0.001*
	25-34	133 (19.5)	51	82	
	35–44	197 (28.9)	91	106	
	45–54	129 (18.9)	48	81	
	54–64	32 (4.7)	8	24	
	65 and above	5 (0.7)	1	4	
Gender	Males	276 (40.5)	131	145	0.937
	Females	405 (59.5)	177	228	
Nationality	Saudi	481 (70.6)	193	288	0.001*
	Non-Saudi	200 (29.4)	115	85	
Region in Saudi Arabia	Central	89 (13.1)	42	47	0.005*
	Eastern	132 (19.4)	65	67	
	Western	221 (32.5)	114	107	
	Northern	37 (5.4)	9	28	
	Southern	202 (29.7)	78	124	
Marital status	Divorced	23 (3.4)	9	14	0.001*
	Married	355 (52.1)	145	210	
	Single	295 (43.3)	154	141	
	Widowed	8 9 (1.2)	0	8	
Education level	Bachelor’s Degree	337 (49.5)	142	195	0.382
	Diploma	115 (16.9)	57	58	
	High school or lower	149 (21.9)	62	87	
	Postgraduate degree	80 (11.7)	47	33	
Work field	Health care field	103 (15.1)	62	41	0.001*
	Not healthcare field	245 (36.0)	97	148	
	Not working	124 (18.2)	48	76	
	Retired	31 (4.6)	7	24	
	Student	178 (26.1)	94	84	
Monthly income	Below 6,000	318 (46.7)	140	178	0.582
	6,000–15,000	231 (33.9)	103	128	
	Above 15,000	132 (19.4)	65	67	
Technological literacy	Competent	168 (24.7)	88	80	0.010*
	Literate	453 (66.5)	18	42	
	Illiterate	60 (8.8)	202	251	
Hours spent on an electronic device	Less than 4 h	161 (23.6)	63	98	0.146
	5–9 h	368 (54.0)	171	197	
	10–15 h	124 (18.2)	57	67	
	More than 15 h	28 (4.1)	17	11	

*Denotes significant *p* values <0.05.

Table 3 presents the results of a chi-square analysis exploring how different socio-demographic factors influenced participants’ views on the safety of robotic-assisted surgery. Age, gender, region, and marital status were significantly associated with safety perception (*p* < 0.05). Younger participants (18–24 years) and males were more likely to consider robotic-assisted surgery safe. Regional differences were also observed, with participants from the Southern region showing the highest confidence in its safety. Marital status was another important factor, as single participants were more likely to believe robotic-assisted surgery was safe compared to married or widowed individuals.

**Table 3:** Chi-square analysis of socio-demographic factors influencing the perception regarding safety of robotic assisted surgery among study participants.

Perception regarding safety of robotic assisted surgery among study participants					
Variable		Total (%)	Yes	No	*p*-value
Age	18–24	185 (27.2)	105	80	0.011*
	25–34	133 (19.5)	60	73	
	35–44	197 (28.9)	108	89	
	45–54	129 (18.9)	82	47	
	54–64	32 (4.7)	11	21	
	65 and above	5 (0.7)	2	3	
Gender	Males	276 (40.5)	182	94	0.001*
	Females	405 (59.5)	186	219	
Nationality	Saudi	481 (70.6)	261	220	0.856
	Non-Saudi	200 (29.4)	107	93	
Region in Saudi Arabia	Central	89 (13.1)	38	51	0.001*
	Eastern	132 (19.4)	70	62	
	Western	221 (32.5)	114	107	
	Northern	37 (5.4)	12	25	
	Southern	202 (29.7)	134	68	
Marital status	Divorced	23 (3.4)	10	13	0.012*
	Married	355 (52.1)	208	147	
	Single	295 (43.3)	149	146	
	Widowed	8 9 (1.2)	1	7	
Education level	Bachelor’s degree	337 (49.5)	179	158	0.257
	Diploma	115 (16.9)	63	52	
	High school or lower	149 (21.9)	89	60	
	Postgraduate degree	80 (11.7)	37	43	
Work field	Health care field	103 (15.1)	64	39	0.102
	Not healthcare field	245 (36.0)	138	107	
	Not working	124 (18.2)	58	66	
	Retired	31 (4.6)	13	18	
	Student	178 (26.1)	95	83	
Monthly income	Below 6,000	318 (46.7)	165	153	0.404
	6,000–15,000	231 (33.9)	133	98	
	Above 15,000	132 (19.4)	70	62	
Technological literacy	Competent	168 (24.7)	97	71	0.268
	Literate	453 (66.5)	36	24	
	Illiterate	60 (8.8)	235	218	
Hours spent on an electronic device	Less than 4 h	161 (23.6)	94	67	0.416
	5–9 h	368 (54.0)	191	177	
	10–15 h	124 (18.2)	70	54	
	More than 15 h	28 (4.1)	13	15	

* Denotes significant *p* values <0.05.

Table 4 presents the results of a logistic regression analysis, which was used to examine the impact of different factors on familiarity with robotic-assisted surgery. The results show that males were less likely to be familiar with robotic-assisted surgery compared to females (OR = 0.689, *p* = 0.02). Saudi participants were significantly more familiar with robotic-assisted surgery than non- Saudis (OR = 1.751, *p* = 0.008).

**Table 4:** Binary logistics regression: effect of socio-demographic factors on familiarity of robotic-assisted surgery.

Variable		OR	CI	*p*-value
Age	18–24	1		
	25–34	3.744	0.233–60.077	0.351
	35–44	1.117	0.073–16.988	0.937
	45–54	1.859	0.125–27.689	0.653
	54–64	1.174	0.080–17.143	0.907
	65 and above	0.709	0.048–10.531	0.803
Gender	Females	1		
	Males	0.689	0.466–0.889	0.02*
Nationality	Non-Saudi	1		
	Saudi	1.751	1.159–2.644	0.008*
Region in Saudi Arabia	Central	1		
	Eastern	1.02	0.569–1.828	0.947
	Western	0.965	0.577–1.613	0.89
	Northern	0.571	0.230–1.414	0.226
	Southern	0.805	0.496–1.312	0.386
Marital status	Divorced	1		
	Married	0.891	0.274–1.34	0.999
	Single	1.012	2.31–0.76	0.999
	Widowed	0.311	0.109–0.826	0.999
Education level	Bachelor’s degree	1		
	Diploma	0.468	0.268–0.816	0.007*
	High school or lower	0.68	0.345–1.341	0.266
	Postgraduate degree	0.451	0.227–0.894	0.022*
Work field	Health care field	1		
	Not healthcare field	0.81	0.232–1.72	0.017*
	Not working	1.221	0.643–2.320	0.542
	Retired	1.511	0.786–2.904	0.215
	Student	0.974	0.287–3.300	0.966
Monthly Income	Below 6,000	1		
	6,000–15,000	1.485	0.950–2.321	0.008*
	Above 15,000	1.495	0.867–2.576	0.148
Technological literacy	Competent	1		
	Literate	1.319	0.918–1.895	0.135
	Illiterate	0.564	0.313–1.018	0.049*
Hours spent on an electronic device	Less than 4 h	1		
	5–9 h	1.155	0.710–1.879	0.561
	10–15 h	1.263	0.861–1.852	0.232
	More than 15 h	1.96	0.847–4.533	0.116

*Denotes significant *p* values <0.05.

Table 5 presents the results of a logistic regression analysis exploring the impact of socio- demographic factors on participants’ perception of robotic-assisted surgery safety. The findings show that males were significantly more likely to consider robotic-assisted surgery unsafe compared to females (OR = 0.504, *p* = 0.001). Participants from the Southern region were more likely to believe robotic-assisted surgery was safe (OR = 2.002, *p* = 0.006). Marital status also had an influence, as single participants were significantly more likely to consider robotic-assisted surgery safe (OR = 10.857, *p* = 0.047).

**Table 5:** Binary logistics regression: effect of socio-demographic factors on perception regarding safety of robotic assisted surgery.

Variable		OR	CI	P-value
Age	18–24	1		
	25–34	1.623	0.160–16.465	0.682
	35–44	0.742	0.077–7.135	0.796
	45–54	1.088	0.117–10.167	0.941
	55–64	1.041	0.113–9.629	0.972
	65 and above	0.59	0.062–5.616	0.646
Gender	Females	1		
	Males	0.504	0.343–0.738	0.001*
Nationality	Non-Saudi	1		
	Saudi	1.171	0.782–1.752	0.443
Region in Saudi Arabia	Central	1		
	Eastern	1.003	0.563–1.787	0.991
	Western	1.214	0.731–2.014	0.453
	Northern	0.58	0.251–1.340	0.202
	Southern	2.002	1.225–3.273	0.006*
Marital status	Divorced	1		
	Married	7.015	0.573–85.811	0.127
	Single	10.857	1.028–114.697	0.047*
	Widowed	5.938	0.545–64.693	0.144
Education level	Bachelor’s degree	1		
	Diploma	1.484	0.855–2.573	0.16
	High school or lower	1.284	0.654–2.522	0.468
	Postgraduate degree	1.911	0.973–3.753	0.06
Work field	Health care field	1		
	Not healthcare field	1.478	0.748–2.921	0.26
	Not working	0.895	0.480–1.668	0.726
	Retired	0.877	0.464–1.655	0.685
	Student	0.417	0.138–1.257	0.12
Monthly income	Below 6,000	1		
	6,000–15,000	1.16	0.750–1.794	0.504
	Above 15,000	0.865	0.507–1.474	0.593
Technological literacy	Competent	1		
	Literate	1.296	0.900–1.868	0.164
	Illiterate	1.324	0.759–2.311	0.324
Hours spent on an electronic device	Less than 4 h	1		
	5–9 h	0.917	0.565–1.490	0.727
	10–15 h	0.781	0.535–1.141	0.201
	More than 15 h	0.584	0.256–1.332	0.152

* Denotes significant *p* values <0.05.

**Figure 3:**
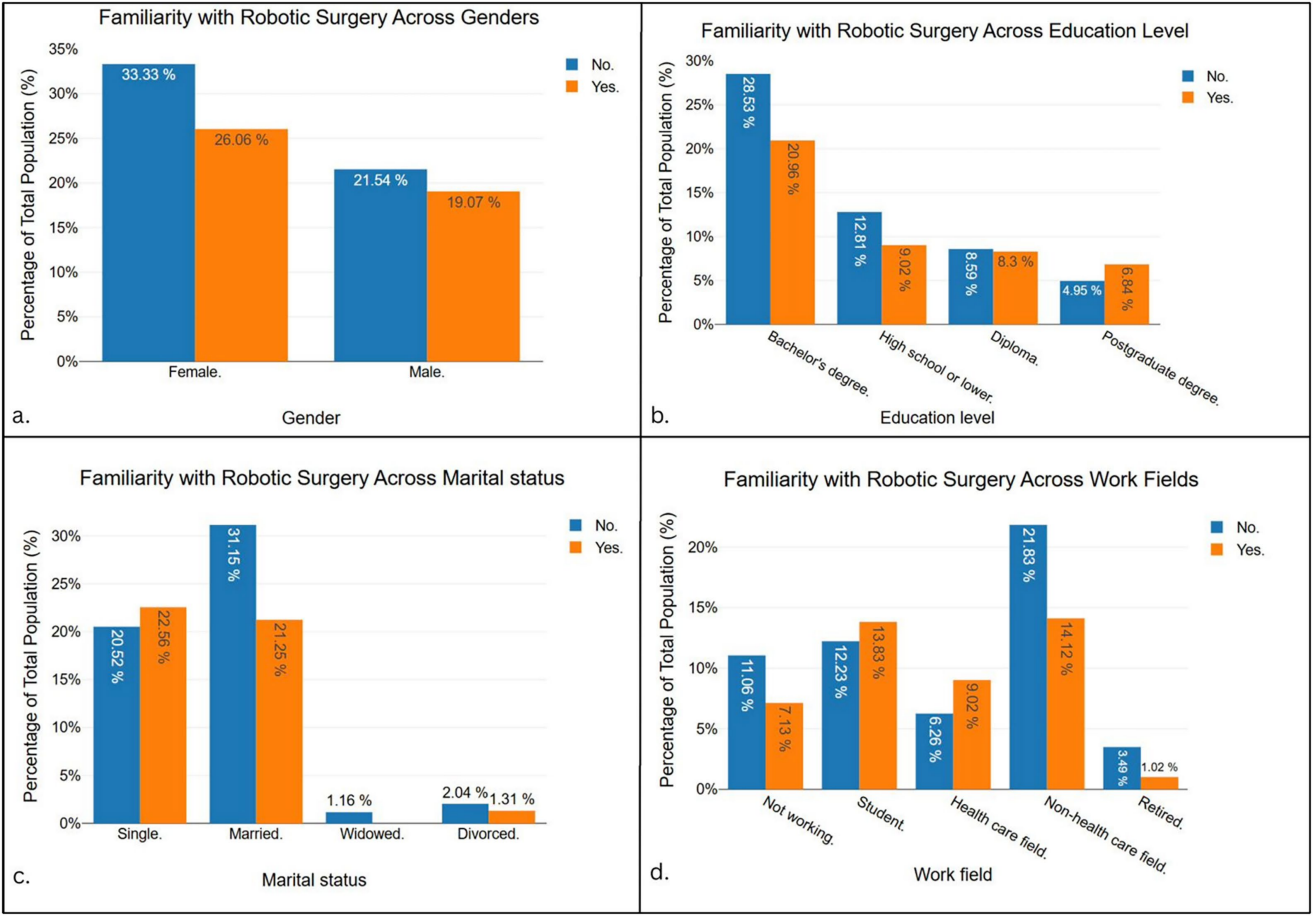
Familiarity with robotic surgery by gender **(a)**, educational level **(b)**, marital status **(c)**, and work field **(d)**. Values are expressed as percentages of the total study population.

Figure 3 shows familiarity with robotic surgery across demographic subgroups. By gender (panel a), a higher proportion of females reported unfamiliarity (33.33%) compared with males (21.54%). Familiarity rates were similar between genders, with 26.06% of females and 19.07% of males indicating awareness. Educational level (panel b) demonstrated that participants with a bachelor’s degree formed the largest group, with 28.53% unfamiliar and 20.95% familiar. Lower familiarity was reported among those with postgraduate education (6.84%) and diploma holders (8.3%). Marital status (panel c) revealed that married participants represented the largest proportion unfamiliar with robotic surgery (31.15%), while single participants reported comparable distributions of unfamiliar (20.52%) and familiar (22.56%). Widowed and divorced groups contributed only small percentages. Work field (panel d) indicated that non-healthcare workers were the largest group unfamiliar with robotic surgery (21.83%), though 14.12% reported familiarity. Students also showed notable familiarity (13.83%), whereas healthcare professionals had more balanced distributions (6.26% familiar vs. 9.02% unfamiliar). Retired participants represented the smallest subgroup.

**Figure 4:**
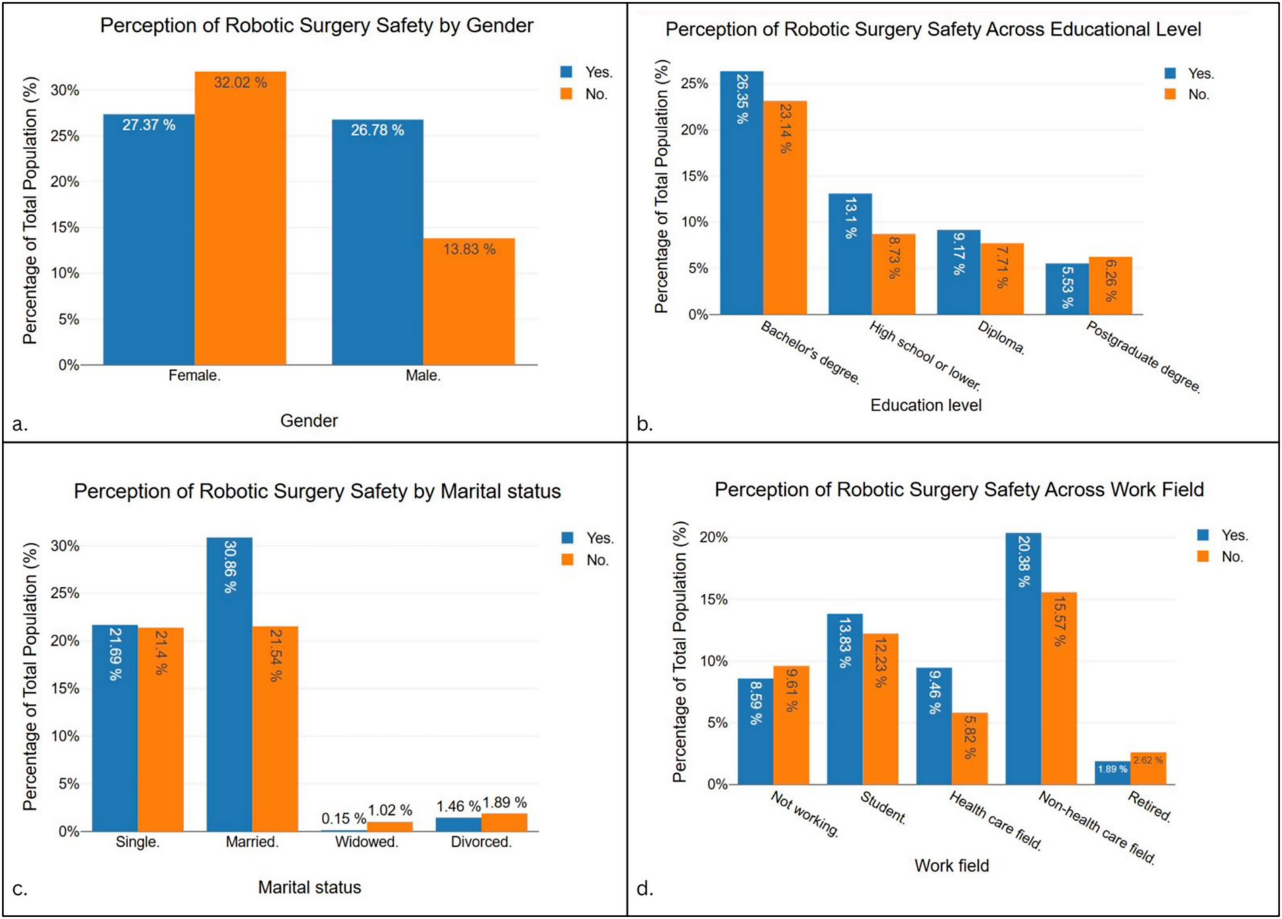
Perceptions of robotic surgery safety stratified by gender **(a)**, educational level **(b)**, marital status **(c)**, and work field **(d)**. Data are presented as percentages of the total study population.

Figure 4 presents the distribution of perceptions regarding the safety of robotic surgery. Among genders (panel a), 27.37% of females and 26.78% of males considered robotic surgery safe, while a larger proportion of females (32.02%) than males (13.83%) considered it unsafe. Across educational levels (panel b), the highest proportion of “safe” responses came from participants with a bachelor’s degree (26.35%), followed by high school or lower (13.1%) and diploma holders (9.17%). Concerns about safety were most common among those with a bachelor’s degree (23.14%). With respect to marital status (panel c), married individuals formed the largest group who considered robotic surgery safe (30.86%), while single participants were evenly split between safe (21.69%) and unsafe (21.4%). Very few responses came from widowed or divorced participants. Work field differences (panel d) showed that healthcare professionals leaned more toward perceiving robotic surgery as safe (9.16% vs. 5.82% unsafe), while non-healthcare workers had higher proportions overall (20.38% safe, 15.57% unsafe). Students also contributed a notable share of positive responses (18.93%), and retired participants were the least represented.

## Discussion

A comprehensive study was done, with a total of 681 participants from various regions of Saudi Arabia, providing key insights regarding public awareness and perception of Robotic-Assisted Surgery (RAS) based on diverse socio-demographic factors, influencing acceptance and concerns. When examined, the study revealed a significant influence by age, gender, nationality, region and marital status among the participants. Higher awareness has been demonstrated by younger individuals, particularly within the age of 18–24 years, which is believed to be due to more insight into recent technological advances. Furthermore, gender differences have been assessed, showing that male groups are more accepting and female groups are more familiar. Also, Saudi citizens exhibited higher familiarity levels compared to non-Saudis, with participants from the Central and Western regions demonstrating greater awareness than those from other areas. Conversely, the Southern region’s members were the most confident in the safety of RAS. Another significant factor was marital status; participants who were single were more familiar with and had a stronger belief in the safety of robotic-assisted surgery than those who were married or widowed, which may have been related to their higher educational attainment, as almost half had a bachelor’s degree.

In line with our findings, which indicate that younger populations are more familiar with/and accepting of RAS, Al Dihan et al. (11) observed that individuals around 21–40 years had more favorable opinions toward robotically assisted approaches. Interestingly, our findings are in line with previous studies conducted in the Middle East. According to Kuwaiti research by Buabbas et al. (4), only roughly 37% of participants had heard of RAS in general, yet many of them thought it improves surgical precision even though they were not familiar with the particular method (4). By contrast, a study from the Western Region of Saudi Arabia found that while 74.5% of participants had heard of RAS, nearly 90% displayed poor knowledge, and only one-third were aware of its availability in the Kingdom (2). In a comparable manner, our findings showed that even though many participants lacked a thorough technical understanding of RAS, they recognized its benefits. Likewise, a study presented by Sultan et al. (12) reported that only about 23% of participants, which are medical students from various colleges in Saudi Arabia, had at least heard of robotic surgery, yet nearly 63% had positive attitudes towards expected outcomes of RAS.

On the other hand, some studies indicate a different picture. Mixed results have been found in multiple studies in relation to gender-based awareness of RAS. For instance, a study by McDermott et al. (13) found that men were slightly more inclined to trust robotic-assisted procedures than women. Our research noted a relatively varied view between genders, which might be attributed to cultural factors and heightened healthcare awareness initiatives in Saudi Arabia. Additionally, AlNaim et al. (14) conducted a study in Saudi Arabia’s Eastern Region, revealing that nearly half of the respondents were entirely unaware of robotic surgery. There were a lot of serious misconceptions, even among people who understood the concept of RAS. This contrasts with our study, which found that RAS was substantially more recognizable to younger and more tech-savvy participants as well as covering all regions of Saudi Arabia, rather than a specific region as has been done in the provided study (14). While our study found that healthcare professionals had greater awareness compared to the public, it also highlighted ongoing concerns about safety and cost, whereas a UAE based study by Barkati et al. (15) emphasized training limitations as a major barrier. The differences suggest that cultural attitudes, exposure to technology, and regional healthcare practices all play a role in shaping how people perceive robotic-assisted surgery.

The COVID-19 pandemic had a major impact on how people in Saudi Arabia view health technology. During this period, telemedicine services and virtual platforms such as the Seha and Mawid apps became widely used and showed high levels of patient satisfaction (16, 17). This experience improved familiarity with digital health tools and built greater trust in technology-based care. It is likely that these shifts also shaped how the public responds to other medical innovations, including robotic-assisted surgery. The higher awareness we observed among younger and more technologically literate groups may, in part, reflect this broader influence of pandemic-driven digital health adoption, which has made the population more open to surgical technologies than before COVID-19.

Our findings highlight the need for targeted education and policy efforts. Older adults, men, and non-Saudi participants demonstrated lower awareness or more frequent misperceptions about RAS, indicating that these groups should be prioritized in awareness campaigns. Public health messages should emphasize safety, effectiveness, and accessibility, and should be delivered through channels that match the target audience, for example, television, and community programs for older adults, and social media platforms for younger populations. Incorporating information on RAS into medical education and wider public health initiatives may also help address misconceptions, build trust, and support broader adoption across the population.

This study included a large and diverse sample with varying educational backgrounds, professional fields, and representation from all regions of Saudi Arabia, providing a wide view of public attitudes toward robotic-assisted surgery. By examining multiple sociodemographic characteristics, the study identified key predictors of awareness and acceptance. Importantly, it assessed both awareness and perception together, whereas many earlier studies evaluated only one aspect. The inclusion of healthcare professionals in the sample also allowed us to highlight potential knowledge gaps within the medical community, offering valuable insight into how levels of familiarity with RAS differ between health workers and the general public. However, there are limitations to consider. The use of convenience sampling, mainly through WhatsApp distribution, may have introduced selection bias by overrepresenting younger and more technologically literate participants, limiting the generalizability of the findings. The sample also predominantly consisted of Saudi nationals, which restricts comparisons across different healthcare systems and cultural contexts. As the data were self-reported, response bias remains possible, with participants potentially overestimating or underestimating their familiarity with RAS. Although the questionnaire was carefully translated and pilot tested, subtle cultural or linguistic differences may still have influenced item interpretation. Another limitation is that while the study assessed perceptions of RAS, it did not explore participants’ willingness to personally undergo such procedures. Finally, the cross-sectional design prevents causal inference. Future studies should consider probability-based sampling, larger and more diverse populations, and qualitative methods to examine individual experiences. Incorporating longitudinal approaches would also help track how awareness and attitudes evolve over time.

The results of this study have important implications from the point of view of clinical practice, healthcare policy, and future research. As the adoption of robotic-assisted surgery is increasing, it is essential to address and resolve public concerns as well as enhance awareness for the same through targeted educational initiatives. Healthcare organizations and policymakers should launch public awareness initiatives to offer individuals straightforward and accessible information regarding the advantages, risks, and safety protocols of robotic-assisted surgery. Moreover, healthcare providers should take a more proactive approach in informing their patients about robotic-assisted procedures. This approach will help build trust while easing concerns about costs and security. Another significant finding of this study is that medical training programs need to better incorporate robotic-assisted surgical teaching. Since our results show that healthcare providers are more familiar with RAS, more should be done to ensure that all medical practitioners have adequate instructions and are exposed to robotic technologies. This could aid in closing the knowledge gap between clinical practice and the general public regarding RAS. Hence, this will ultimately improve patient related outcomes and increase their confidence in robotic-assisted procedures.

Future research should extend beyond assessing awareness to examine the extent to which individuals are willing to actively engage with robotic-assisted surgery. This includes evaluating patient readiness to use digital platforms for pre-operative education, to contribute post-operative data through patient-reported outcome measures (PROMs), and to participate in outcome registries. It will also be important to monitor how perceptions of robotic surgery evolve over time as the technology becomes more widely implemented, since repeated exposure may foster greater trust and acceptance (18). Qualitative studies exploring the experiences of patients undergoing robotic procedures could provide valuable insight into the factors shaping satisfaction and acceptance. Likewise, examining the perspectives of surgeons and other healthcare professionals would help identify operational and training challenges that may limit broader integration. Addressing these research gaps would support the development of targeted education initiatives and patient-centered strategies, ultimately improving public trust and contributing to the wider and more effective adoption of robotic-assisted surgery.

## Conclusion

This study shows that awareness and perceptions of robotic-assisted surgery in Saudi Arabia are shaped by factors such as age, gender, nationality, and education. Younger and more technologically literate individuals reported greater familiarity, while concerns about cost and safety were common among other groups. These results point to the need for targeted public awareness campaigns, integration of RAS into medical curricula, and culturally sensitive communication strategies to build trust and acceptance. Policymakers should ensure that accurate and accessible information is available to the public as RAS becomes more widely adopted in the Kingdom.

## Data Availability

The original contributions presented in the study are included in the article/supplementary material, further inquiries can be directed to the corresponding author/s.
