# Correction: Public awareness and perception of robotic-assisted surgery: a cross-sectional analysis of sociodemographic influences

**DOI:** 10.3389/fpubh.2025.1746508

**Published:** 2025-11-26

**Authors:** Husna Irfan Thalib, Khadijah Tahir, Ayesha Shrouq Amin, Zahra Hussein Alabdrabalrasol, Sarah Kaleem Ather, Bader Mahmoud Almurad, Sara Ahmed Al Nawajha, Ahmed A. Elshora, Mohammed Ridha Algethami, Noor Ahmad Shaik, Salem Sroor Al Bagmi

**Affiliations:** 1 College of Medicine and Surgery, Batterjee Medical College, Jeddah, Saudi Arabia; 2 Department of Surgery, General Medicine Program, Batterjee Medical College, Jeddah, Saudi Arabia; 3 Department of Family and Community Medicine, College of Medicine, University of Jeddah, Jeddah, Saudi Arabia; 4 Department of Genetic Medicine, Faculty of Medicine, King Abdulaziz University, Jeddah, Saudi Arabia; 5 Department of Health Information Management, Prince Sultan Military College of Health Sciences, Dammam, Saudi Arabia

**Keywords:** robotic-assisted surgery, public awareness, Saudi Arabia, healthcare adoption, perception

Affiliation “Department of Genetic Medicine, Faculty of Medicine, King Abdulaziz University, Jeddah, Saudi Arabia” was omitted for author Noor Ahmad Shaik. This affiliation has now been added for author Noor Ahmad Shaik.

Author Noor Ahmad Shaik was erroneously assigned to affiliation “Department of Family and Community Medicine, College of Medicine, University of Jeddah, Jeddah, Saudi Arabia”. This affiliation has now been removed for author Noor Ahmad Shaik.

Affiliation “Department of Health Information Management, Prince Sultan Military College of Health Sciences, Dammam, Saudi Arabia” was omitted for author Salem Sroor Al Bagmi. This affiliation has now been added for author Salem Sroor Al Bagmi. Author Salem Sroor Al Bagmi was erroneously assigned to affiliation “Department of Genetic Medicine, Faculty of Medicine, King Abdulaziz University, Jeddah, Saudi Arabia”. This affiliation has now been removed for author Salem Sroor Al Bagmi.

The original version of this article has been updated.

